# Preoperative Staging in Oral Cavity Cancer: Nationwide Practice and Concordance With Pathology

**DOI:** 10.1111/odi.70082

**Published:** 2025-08-31

**Authors:** Hanneke Doremiek van Oorschot, Julie Maria Leonardus Sijmons, Jose Angelito Hardillo, Robert Jan Baatenburg de Jong, Dutch Head and Neck Audit ‐ Oral Cavity Collaborator Group, Robert J. J. van Es, Guido B. van den Broek, Robert P. Takes, Gyorgy B. Halmos, Dominique V. C. de Jel, Jan‐Jaap Hendrickx, Richard Dirven, Martin Lacko, Lauretta A. A. Vaassen, Alexander J. M. van Bemmel, Reinoud Klijn, Marjolijn A. E. Oomens, Hossein Ghaeminia, Leonora Q. Schwandt, Christiaan A. Krabbe, Annemarie Vesseur, Rolf Bun, Thomas J. W. Klein Nulent, Jeroen C. Jansen

**Affiliations:** ^1^ Department of Otorhinolaryngology and Head and Neck Surgery Erasmus Medical Center Cancer Institute Rotterdam the Netherlands; ^2^ Scientific Bureau Dutch Institute for Clinical Auditing Leiden the Netherlands; ^3^ Department of Surgery Amsterdam University Medical Center, University of Amsterdam Amsterdam the Netherlands; ^4^ Treatment and Quality of Life Cancer Center Amsterdam Amsterdam the Netherlands

**Keywords:** head and neck cancer, hospital variation, locoregional staging, oral cavity cancer, TNM staging system

## Abstract

**Objectives:**

The clarity of TNM‐classification for oral cancer has a direct impact on healthcare resource allocation, treatment decisions, morbidity, and clinical outcomes. However, possible TNM ambiguity between hospitals exists due to the broad range of available diagnostics. Therefore, this study aims to assess current practice variation in preoperative staging for oral cavity cancer.

**Materials and Methods:**

All patients who underwent primary oral cavity cancer resection in the Netherlands between 2018 and 2021 were selected from the Dutch Head and Neck Audit database. Preoperative staging (cTN) was compared to definitive pathology staging (pTN) as the gold standard for assessing concordance at national and hospital levels.

**Results:**

Disease stage was upstaged in 27.5% of the patients. For T‐classification analysis, 2458 patients were included. Accuracy for T‐classification categories was 85.2%–93.1%, but significant hospital variation in overstaging and understaging was observed. For N‐classification analysis, 1746 patients were included. Preoperative assessment of node involvement missed metastasis in 25.1% of the cN0 patients, resulting in 77.3% accuracy.

**Conclusion:**

Significant preoperative understaging of oral cancer calls the attention to the difficulties of the diagnostics of oral cancer. Although the accuracy of preoperative staging in oral cavity cancer is high, significant differences between hospitals were observed.

## 1 Introduction

Preoperative locoregional staging is the backbone of algorithms for the diagnosis and treatment of oral cavity cancer (OCC, Brierley et al. 2016). More importantly, tumour stage is the most crucial prognostic factor in OCC (van der Schroeff and Baatenburg de Jong 2009; Weimar et al. 2018). This underscores the fundamentality of correct tumour staging. Yet previous studies have reported discordance between cTNM and pTNM in OCC (Kreppel et al. 2016; Choi et al. 2017; Biron et al. 2013; Kılıç et al. 2018). When clinical and pathological classifications differ, this casts doubt on treatment plans and patient prognosis. In addition, inclusion criteria for clinical trials regularly include cTNM. Therefore, trial outcomes may be influenced when initial cTNM staging is incorrect (van der Schroeff and Baatenburg de Jong 2009). However, these outcomes eventually contribute to treatment guidelines.

The patient's initial treatment plan follows from the cTNM. In head and neck cancer (HNC), a broad selection of diagnostic tools is available for assessing the primary tumour site, lymph node involvement and distant metastasis (Antoniou et al. 2014; Mupparapu and Shanti 2018). Following international guidelines, the workup can include physical examination, Magnetic Resonance Imaging (MRI), Computed Tomography (CT), intraoral ultrasonography, orthopantomogram, ultrasound with fine needle aspiration cytology (UGFNAC), fluorodeoxyglucose positron emission tomography (FDG‐PET) CT/MRI or combinations of these modalities (Mupparapu and Shanti 2018; Federatie Medisch Specialisten 2014). In practice, hospitals have incorporated local diagnostic protocols. As diagnostic tool accuracy differs, interobserver disagreement, under‐ and overstaging rates will vary between centres (Antoniou et al. 2014; Marcello Scotti et al. 2022; Locatello et al. 2020; Brandão Neto et al. 2018; Subramaniam et al. 2022; Sun et al. 2015). This raises the question of to what extent cTNM classification from different hospitals can be interpreted similarly. However, stage unambiguity is assumed in multicentre or national research and evaluation of quality of care (Takes et al. 2020).

Therefore, this study aimed to assess the concordance of preoperative and pathological OCC staging according to TNM in the Netherlands. Accuracy and practice variation of pathological staging were analysed nationally and at hospital level. Insight into the extent of cTNM‐pTNM discrepancy is necessary to improve the quality of tumour staging.

## 2 Material and Methods

### 2.1 Study Design

Data was extracted from the Dutch Head and Neck Audit (DHNA) database. The DHNA is one of 26 quality registries administered at the Dutch Institute for Clinical Auditing (Beck et al. 2020). HNC treatment in the Netherlands is executed in fourteen hospitals, seven of which are academic hospitals. All HNC hospitals have contributed to the DHNA since 2018, in which all patients with a first primary head and neck tumour are registered (van Overveld et al. 2018). In 2022, OCC incidence in the Netherlands was ~1000 (Integraal Kankercentrum Nederland 2023). Health insurance is compulsory and includes head and neck cancer (HNC) care. This study did not require obtaining informed consent from patients or medical ethics committee's approval under Dutch Civil Law (Article 7:458). This legislation applied because registration data was reused and did not include directly identifiable data.

### 2.2 Population

Patients who presented with first primary OCC between January 1, 2018 and December 31, 2022, were included if surgically treated with curative intent. Patients were excluded in case of neoadjuvant therapy. For N‐classification analysis, patients were included when (elective) neck dissection (END) or sentinel lymph node biopsy (SLNB) was performed. When included, the following variables were extracted: age, gender, body mass index (BMI), tumour histology, the time interval between multidisciplinary team (MDT) meeting and surgery, adjuvant therapy, treating hospital and TNM‐classification following the 8th edition of the Union for International Cancer Control TNM Classification (Brierley et al. 2016). The BMI was included because evidence exists that this variable may influence the diagnostic performance of ultrasound and MRI for node involvement (Macaione et al. 2020; Chen et al. 2022).

### 2.3 Guidelines and Definitions

In the DHNA, clinical TNM classification is based on preoperative diagnostic workup and MDT decision. Local diagnostic protocols differ between the HNC hospitals, but all employ dedicated head and neck radiologists (Dutch Head and Neck Society 2017). The pathology TNM classification is based on the postoperative pathology report and MDT decision. The resection specimen was assessed by a dedicated HNC pathologist.

### 2.4 Statistical Analysis

Based on the distribution, data is presented as a median with interquartile range (IQR). Categorical variables are presented as record counts with percentages. The preoperative staging (cTN) was compared to definitive pathology staging (pTN) as the gold standard to assess concordance at national and hospital levels. Comparison between the clinical and pathological disease stages was performed for the N‐analysis cohort because data on N‐classification was missing for some patients in the T‐analysis cohort. Understaging was defined as a clinical T/N‐classification lower than the pathological T/N‐classification. Vice versa, overstaging was defined as a clinical T/N‐classification higher than the pathological T/N‐classification. Confusion matrices were created with the cTNM classification as predicted values and pTNM classification as actual values. Comparisons were made for the classification of (1) T1 versus T2‐4, (2) T1‐2 versus T3‐4, (3) T4 versus T1‐3 and (4) N0 versus N1‐3.

The point estimates accuracy, sensitivity, specificity, positive predictive value (PPV) and negative predictive value (NPV) were calculated with 95% confidence intervals. To determine the variation in point estimates between hospitals, the mean percentages, including the minimum and maximum for each estimate, were calculated. In addition, a violin graph was plotted to show the distribution of the point estimates in the hospitals. Univariable logistic regression analyses were performed to assess whether effect estimates significantly differ between hospitals. Reported *p* values were considered significant if below 0.05. Analysis was performed using the R software system for statistical computing (version 4.2).

## 3 Results

### 3.1 Study Population

**FIGURE 1:**
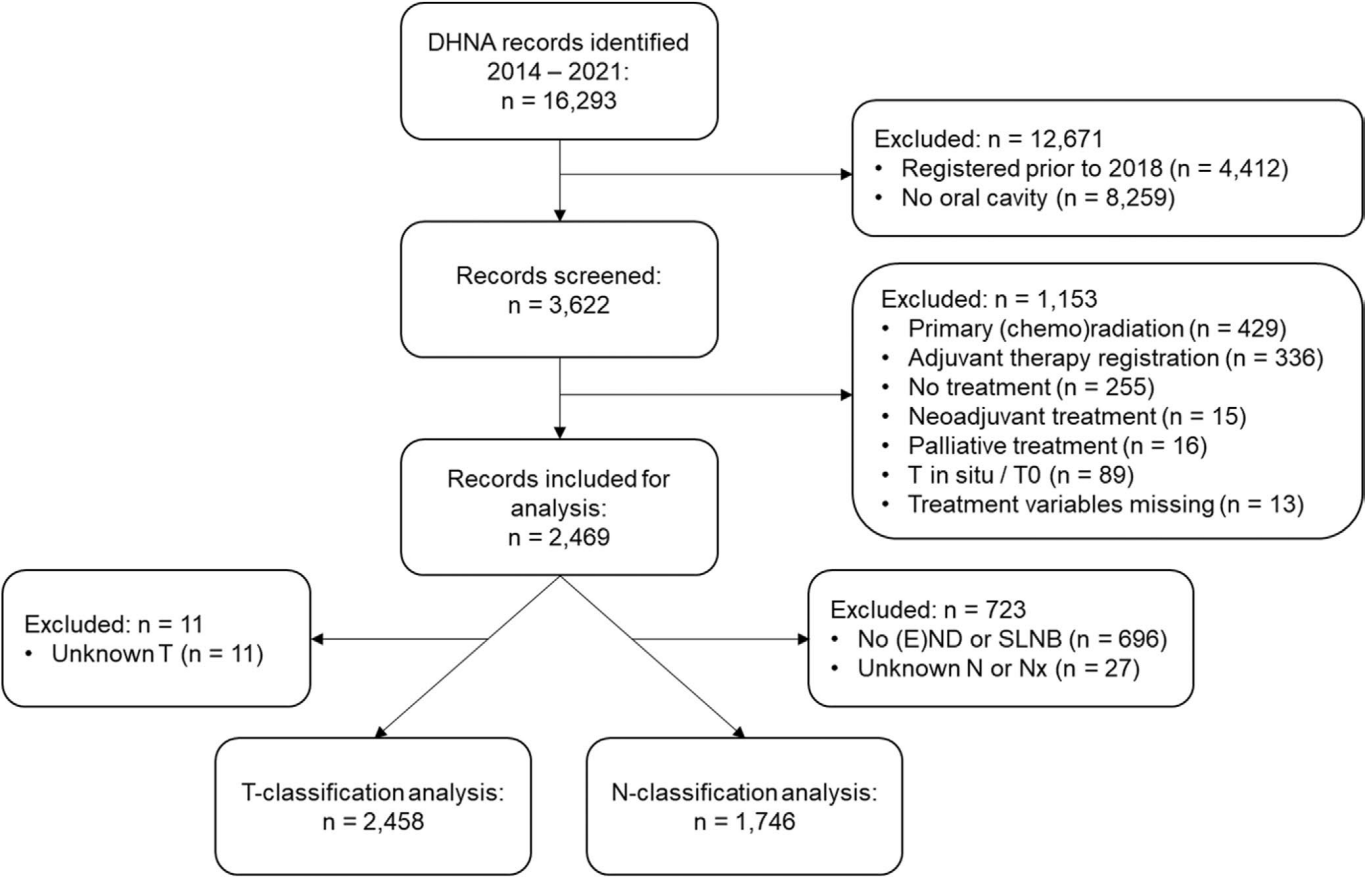
Flow chart for study inclusion. DHNA, Dutch head and neck audit; (E) ND, (elective) neck dissection; SLNB, sentinel lymph node biopsy.

A total of 2469 OCC patients were selected from the DHNA database (Figure 1). Completeness of registration was high for T‐classification (99.8%): 2458 patients were included for T‐analysis. For N‐analysis, 1746 patients were included, with 98.5% completeness of registration in patients who underwent (E)ND or SLNB. For T‐ and N‐classification, patients were predominantly male (54.6%/56.1%) with a median age of 66/68 years and a BMI of < 25 kg/m^2^ in 43.0%/48.1% (Table 1). Academic hospitals treated 71.1% to 74.0% of the patients. Pathological presence of lymph node metastasis varied from 9.0% in pT1 tumours to 54.5% in pT3 tumours (Table 2). There was only one case of distant metastasis in the pT3 group.

**TABLE 1:** Demographic characteristics for analysis of tumour staging (T‐analysis) and node involvement (N‐analysis).

	T‐analysis	N‐analysis
Characteristic	*n* = 2458	*n* = 1746
Sex		
Female	1115 (45.4%)	767 (43.9%)
Age		
Median years (IQR)	68 (59, 76)	66 (58, 74)
BMI		
< 25 kg/m^2^	1057 (43.0%)	839 (48.1%)
≥ 25 kg/m^2^	1196 (48.7%)	872 (49.9%)
Unknown	205 (8.3%)	35 (2.0%)
Histology		
Squamous cell carcinoma	2135 (86.9%)	1590 (91.1%)
Non‐squamous cell carcinoma	168 (6.8%)	43 (2.5%)
Unknown	155 (6.3%)	113 (6.5%)
Time interval MDT to start treatment		
Median days (IQR)	15 (10, 21)	16 (11, 23)
Unknown	223 (9.5%)	31 (1.8%)
Treatment		
Surgery	1593 (64.8%)	970 (55.6%)
Surgery and adjuvant therapy	865 (35.2%)	776 (44.4%)
Hospital type		
Academic hospital	1748 (71.1%)	1292 (74.0%)
Non‐academic hospital	710 (28.9%)	454 (26.0%)
Hospital volume		
N (minimum–maximum)	58–363	40–272
Year of registration		
2018	550 (22.4%)	390 (22.3%)
2019	615 (25.0%)	441 (25.3%)
2020	624 (25.4%)	447 (25.6%)
2021	669 (27.2%)	468 (26.8%)

Abbreviations: BMI, body mass index; IQR, interquartile range; MDT, multidisciplinary team.

**TABLE 2:** pT‐classification with corresponding pNM‐classification.

pT classification	Number of patients	
*n* = 2458	*n*	%
pT1	971	
pN0	457	47.1
pN1	35	3.6
pN2	32	3.3
pN3	20	2.1
pNx/pN unknown	427	44.0
pM0	98	10.1
pM1	—	—
pMx/pM unknown	873	89.9
pT2	671	
pN0	368	54.8
pN1	86	12.8
pN2	78	11.6
pN3	40	6.0
pNx/pN unknown	99	14.8
pM0	88	13.1
pM1	—	—
pMx/pM unknown	583	86.9
pT3	352	
pN0	141	40.1
pN1	51	14.5
pN2	61	17.3
pN3	80	22.7
pNx/pN unknown	19	5.4
pM0	51	14.5
pM1	1	0.3
pMx/pM unknown	300	85.2
pT4	464	
pN0	207	44.6
pN1	50	10.8
pN2	88	19.0
pN3	82	17.7
pNx/pN unknown	37	8.0
pM0	61	13.1
pM1	—	—
pMx/pM unknown	403	86.9

### 3.2 Diagnostic Reliability of Disease Stage

Clinical disease stage remained unchanged compared to the pathological disease stage in 60.5% of the patients (*n* = 1756, Figure 2a). The disease stage was upstaged in 27.5% and downstaged in 12.0%. Upstaging was highest in stage III disease (50.2%) and lowest in stage I disease (35.4%). Downstaging occurred in 19.6% of stage II, 10.9% of stage III and 14.3% of stage IV disease.

**FIGURE 2:**
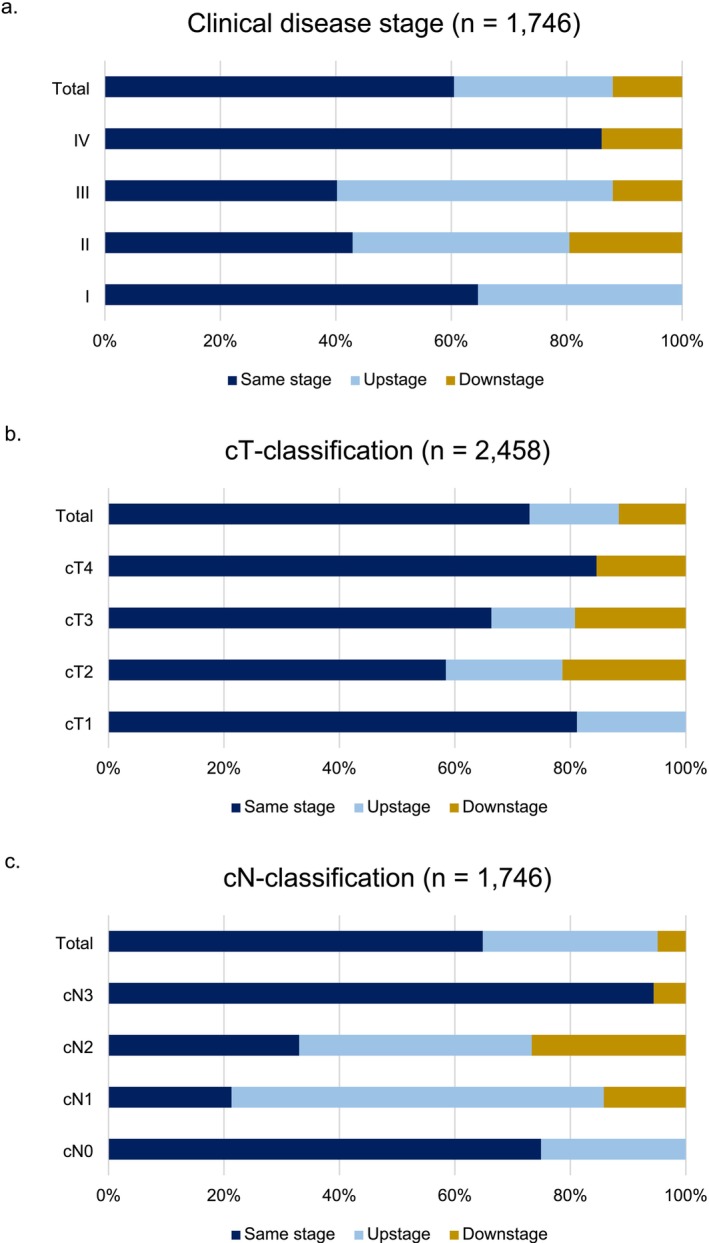
Comparison of clinical and pathological disease stage and TN‐classification. (a) clinical disease stage. (b) cT‐classification. (c) cN‐classification.

### 3.3 Diagnostic Reliability of T‐Classification

**FIGURE 3:**
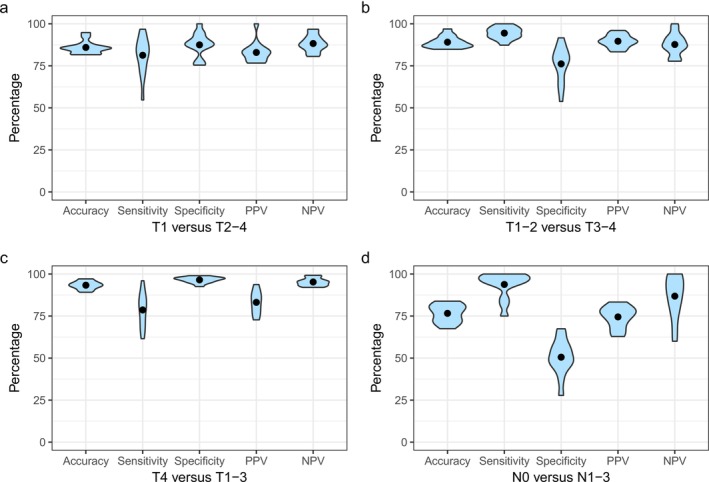
Violin graph showing the distribution of mean percentages (range) of point estimate variables per hospital in the Netherlands for (a) T1 versus T2 to 4, (b) T1‐2 versus T3 to 4, (c) T4 versus T1 to 3, and (d) N0 versus N1 to 3. NPV, negative predictive value; PPV, positive predictive value.

Overall, the concordance rate of clinical T‐classification was 72.9%, with 15.5% upstaging and 11.6% downstaging (Figure 2b). Accuracy for the distinction between T1 and T2‐4 was 85.2%, with the other point estimates varying from 81.1% to 87.9% (Table 3A, Figure 3a). Here, hospital variation was significant for sensitivity, specificity, and NPV. For differentiating between cT1‐2 and cT3‐4, accuracy was 88.7% and specificity was 77.3%, indicating significant hospital variation (Table 3B, Figure 3b). Concordance for cT4 resulted in 93.1% accuracy, with significant hospital variation for sensitivity and NPV (Table 3C, Figure 3c).

**TABLE 3:** Concordance of preoperative and pathology T‐classification.

Preoperative, cT‐classification	Pathology, pT‐classification				
	pT1	pT2	pT3	pT4	Total
cT1	792	152	16	16	976
cT2	162	443	109	44	758
cT3	8	49	197	43	297
cT4	9	27	30	361	427
Total	971	671	352	464	2458

Point estimate	%	95% CI	Hospital variation		
			Min %	Max %	*p* *
A. Reliability of preoperative staging in differentiating pT1 from pT2‐4					
Accuracy	85.2	83.8, 86.6	81.7	94.8	0.29
Sensitivity	81.6	79.0, 84.0	54.7	96.8	< 0.001
Specificity	87.6	85.8, 89.3	75.4	100.0	< 0.001
PPV	81.1	78.5, 83.6	76.7	100.0	0.39
NPV	87.9	86.2, 89.5	80.7	96.8	0.01
B. Reliability of preoperative staging in differentiating pT1‐2 from pT3‐4					
Accuracy	88.7	87.4, 89.9	84.9	96.9	0.15
Sensitivity	94.3	93.1, 95.4	87.3	100.0	0.001
Specificity	77.3	74.3, 80.2	53.9	91.7	0.008
PPV	89.3	87.8, 90.7	83.3	96.1	0.10
NPV	87.2	84.5, 89.5	77.8	100.0	0.14
C. Reliability of preoperative staging in differentiating pT4 from pT1‐3					
Accuracy	93.1	92.1, 94.1	89.2	97.1	0.20
Sensitivity	77.8	73.7, 81.5	61.5	96.0	0.05
Specificity	96.7	95.8, 97.4	92.6	99.0	0.16
PPV	84.5	80.8, 87.8	72.7	93.8	0.55
NPV	94.9	93.9, 95.8	92.0	99.2	0.05

Abbreviations: CI, confidence interval; NPV, negative predictive value; PPV, positive predictive value.

* *p* values were calculated using univariable logistic regression analysis with the point estimates as dependent variables and the hospitals as an independent variable.

### 3.4 Diagnostic Reliability of N‐Classification

Occult lymph node metastasis was detected in 25.1% of the cN0 patients (Figure 2c). Pathology did not yield positive nodes in 6.2% of the cN1‐3 patients. Preoperative N‐assessment indicated a sensitivity, specificity, PPV, NPV and accuracy of 93.8%, 52.2%, 74.9%, 84.7% and 77.3%, respectively (Table 4, Figure 3d). Hospital variation was significant for sensitivity, specificity and NPV.

**TABLE 4:** Concordance of preoperative and pathology N‐classification.

Preoperative, cN‐ classification	Pathology, pN‐classification				
	pN0	pN1	pN2	pN3	Total
cN0	989	163	118	50	1320
cN1	24	36	62	47	169
cN2	39	20	73	89	221
cN3	2	0	0	34	36
Total	1054	219	253	220	1746

Point estimate	%	95% CI	Hospital variation		
			Min %	Max %	*p* *
A. Reliability of preoperative staging in differentiating N0 from N1‐3					
Accuracy	77.3	75.3, 79.2	67.5	83.8	0.18
Sensitivity	93.8	92.2, 95.2	75.0	100.0	< 0.001
Specificity	52.2	48.4, 55.9	27.8	67.4	0.03
PPV	74.9	72.5, 77.2	62.9	83.3	0.18
NPV	84.7	81.0, 88.0	60.0	100.0	< 0.001

Abbreviations: CI, confidence interval; NPV, negative predictive value; PPV, positive predictive value.

* *p* values were calculated using univariable logistic regression analysis with the point estimates as dependent variables and the hospitals as an independent variable.

## 4 Discussion

This population‐based cohort study assessed the concordance of preoperative and pathological OCC staging in HNC centres in the Netherlands, analysing 2469 cases. Overall, the disease stage remained unchanged in over half of the patients but was upstaged in over a quarter. The accuracy of cTN‐classification was high nationally and did not indicate hospital variation. Some point estimates did, however, indicate significant differences between hospitals.

### 4.1 T‐Classification

Our reported overall T‐classification concordance rate and accuracy comply with previous literature. In two single‐centre studies on 252 and 392 OCC patients, T‐classification remained consistent at 61.7% and 87.3%, respectively (Kreppel et al. 2016; Choi et al. 2017). A multicentre study reported that cT was unchanged in 68.1% of 379 OCC patients (Biron et al. 2013). Overall survival was not significantly impacted by up‐ or downstaging in these studies (Kreppel et al. 2016; Biron et al. 2013). In one study, upstaging was explained by the underestimation of surface dimension and deep invasion through tongue muscles (Kreppel et al. 2016). A study using data from the National Cancer Database in the United States found 72.4% T‐classification concordance in 9110 OCC cases. They described a significant decrease in overall survival for upstaged patients of all tumour stages except clinical stage IVB (Kılıç et al. 2018).

The outcomes we have obtained for T‐classification concordance and hospital variation are impacted by the following factors. First, in case of uncertainty about a patient's TNM‐classification during the MDT meeting, the case will be downstaged. This could account for a portion of the reported understaging rates. Secondly, Dutch hospitals adhere to local protocols with varying diagnostic modalities of choice. For instance, seven Dutch HNC hospitals standardly subject all OCC‐suspected patients to imaging (one hospital CT, six hospitals MRI). Good diagnostic accuracy for MRI and CT has been reported regarding tumour thickness, depth of invasion (DOI), and detection of bone invasion in OCC (Marcello Scotti et al. 2022; Brandão Neto et al. 2018). Current guidelines permit hospitals to opt for either imaging or solely physical examination in small OCC tumours (Mupparapu and Shanti 2018). Therefore, the other seven HNC hospitals perform imaging for cT3‐4, and in cT2 tumours only when in doubt about the DOI. Two hospitals use intraoral ultrasound for soft‐tissue sites in early OCC (Klein Nulent et al. 2018). This variation could contribute to the observed significant hospital variation in sensitivity and specificity for preoperative cT1 and cT1‐2 distinction. Some hospitals have standardly incorporated PET‐CT in their diagnostic protocols, but this occurred after our study period. The reported reliability of clinical T4‐classification in our study suggested an understaging of bone marrow invasion. The significant hospital variation in sensitivity and NPV may stem from local preferences for CT or MRI in tumour classification. For this study, all histological types of OCC were included. This approach stems from the belief that correct tumour staging should not depend on the histological subtype of OCC. In additional analysis, accuracy for all four comparisons was not significantly different for squamous cell carcinoma versus other histological types (Table S1).

### 4.2 N‐Classification

The presence of lymph node metastasis is considered the most crucial prognostic factor in OCC (Mupparapu and Shanti 2018). For N‐classification, previous studies have both confirmed and disputed the association between overstaging and survival (Kreppel et al. 2016; Choi et al. 2017). The American Cancer Database study found 28.5% discordance for N‐classification, primarily due to postoperative upstaging (Kılıç et al. 2018). Our study confirms this finding.

Clinical staging of the neck starts with physical and ultrasound examinations in all Dutch hospitals. Fine needle aspiration cytology is performed for suspected nodes on ultrasound (Mupparapu and Shanti 2018). Additional imaging is not routinely performed for cN0 patients; however, if the MRI or CT scan used for primary tumour staging also includes the neck, it will provide Supporting Information. A systematic review comparing CT and MRI to detect cervical lymph node metastasis found higher specificity for MRI (81%) and higher sensitivity for CT (77%, Sun et al. 2015). From cN2 and higher, MRI, PET‐CT and chest CT are used to assess node involvement and distant metastasis (Mupparapu and Shanti 2018). In a multicentre trial, the NPV of FDG‐PET CT for N0 clinical neck was a promising 86% (Subramaniam et al. 2022). Despite the comprehensive selection of tools for node assessment, occult metastasis in OCC remains a concern. In our cohort, occult metastasis manifested after radiological examination in 25.1% of cN0 patients, whereas existing literature reports a range of 15%–444% (Choi et al. 2017; Mupparapu and Shanti 2018). As clinical or radiological N‐classification alone is inadequate, SLNB and END are crucial for cT2‐4 classification. In the Netherlands, SLNB is executed by 12 hospitals, whereas the other two perform END. These surgical procedures have been installed as an additional diagnostic tool after clinical staging to achieve the best survival outcomes for patients, since lymph node involvement significantly impacts survival rates (Mupparapu and Shanti 2018).

Precaution regarding occult metastasis is reflected in the high sensitivity and low specificity of our study. The overall sensitivity and NPV for distinguishing cN0 from cN1‐3 were high, yet significant hospital variation in overstaging existed. Again, local imaging preferences could play a role. Hospital protocols for regional metastasis differ, but UGFNAC is still the most used across the Netherlands. Information for N‐classification is also obtained from the MRIs that are standardized for tumour staging in half of Dutch hospitals. For cN2‐3 tumours, many centres also conduct FDG‐PET CT/MRI to assess M‐classification.

### 4.3 Strengths and Limitations

This study included a substantial sample size from a national database. The unique character of the DHNA database enabled us to investigate hospital variation in locoregional staging. DHNA data is collected by trained registrars and subjected to data verification and validation processes (Beck et al. 2020). Nonetheless, this study also has some limitations. DHNA data is primarily collected for auditing instead of scientific purposes and only includes patients as treated, thus creating indication bias. Unfortunately, data on the specific performed imaging modalities, scan type, slice thickness and radiologist's expertise are not included. Therefore, the impact of the individual scans on point estimates or over time could not be investigated. Changes in diagnostic protocols possibly introduced bias as this improved tumour assessment and moved patients to another TNM classification (van der Schroeff and Baatenburg de Jong 2009). Moreover, local practice regarding sample handling and interobserver variability in pathologic evaluation impacts the pTNM classification (Morbini et al. 2025). The extent of hospital variation for diagnostic accuracy in HNC patients treated non‐surgically could not be investigated. For this population, the consequences of incorrect TNM classification could be more severe as classification cannot be rectified after surgery. We could not investigate PET‐CT performance on TN classification, as the routine use has been incorporated after our study period.

### 4.4 Implications and Future Prospects

TNM unambiguity is essential for reliable multicentre research and evaluation of quality of care. Therefore, hospital variation in TNM concordance should be accounted for in sample size calculation for multicentre collaboration, grouped national analysis and study inclusions. During the uncertain diagnostic and therapeutic periods, managing patient expectations is vital. Clinicians should, therefore, be aware of the extent of OCC stage discordance when delivering a diagnosis. Theoretically, our results could be influenced by the hospital level; however, the additional regression analysis did not find differences in staging accuracy between academic and non‐academic hospitals (Table S1). The BMI was included because breast cancer studies have shown that adipose tissue affects the diagnostic performance of ultrasound and MRI (Macaione et al. 2020; Chen et al. 2022). In our study, the T‐ and N‐classification accuracy was not influenced by BMI (Table S1). Since patient‐level diagnostic test data were not available, a possible association cannot definitively be ruled out. Therefore, additional studies are recommended to explore this relationship in the context of head and neck cancer.

The balance between under‐ and overstaging in oncologic care is especially precarious. Misjudgment in tumour delineation due to up‐ or downstaging may lead to resection with inadequate margins or the unnecessary sacrifice of tissue (Choi et al. 2017). Moreover, adjuvant therapy is often considered for T3‐4 tumours. Incorrect classification may result in unnecessary preoperative referrals to a radiation oncologist or dentist for dental clearance. Overstaging of the neck could result in unnecessary neck dissections and consecutive morbidity. Moreover, incorrect classification can cause treatment delays if logistics are initiated after pathological confirmation. Both treatment delay and inadequate resections are associated with worse survival outcomes (Graboyes et al. 2019; Sunkara et al. 2023). Unfortunately, our data did not enable us to research these consequences. Studies such as this can help monitor up‐ and downstaging rates across hospitals. Cooperative research with all HNC hospitals should focus on comparing local diagnostic work‐up protocols. Finally, the optimal work‐up can possibly be identified to minimise exposure to more aggressive treatments or missed opportunities for appropriate interventions.

As healthcare expenses rise and planetary health issues increase, clinicians are advised to be discerning in their utilisation of medical resources, including imaging modalities. Within the DHNA, hospitals receive performance feedback to support benchmarking and prompt local analysis for continuous improvement. Individual hospital results from this study have been reported to all participating centres and discussed in DHNA meetings. Follow‐up studies in the performance of separate imaging modalities and the treatment consequences of incorrect staging have been instigated. Apart from accuracy measures, financial aspects and sustainability are important criteria to design a future‐proof national diagnostic pathway.

## 5 Conclusion

The reliability of preoperative locoregional staging for OCC in the Netherlands was high, but differences at the hospital level were observed. TNM unambiguity across hospitals is essential to minimise patient under‐ and overtreatment and facilitate reliable evaluation of quality of care. Discrepancies between clinical and pathology TN‐classification should be considered in multicentre research. With increasing costs and environmental impacts, prudent use of imaging and other medical resources is recommended for clinicians. Future collaborative research projects should therefore focus on coordination of diagnostic pathways on a national level.

## Author Contributions

**Hanneke Doremiek van Oorschot:** conceptualization, investigation, writing – original draft, methodology, visualization, writing – review and editing, formal analysis. **Julie Maria Leonardus Sijmons:** conceptualization, investigation, writing – original draft, methodology, visualization, writing – review and editing, formal analysis. **Jose Angelito Hardillo:** conceptualization, supervision, data curation, writing – review and editing, writing – original draft. **Robert Jan Baatenburg de Jong:** conceptualization, writing – review and editing, data curation, supervision. **Robert J. J. van Es:** data curation, writing – review and editing. **Guido B. van den Broek:** writing – review and editing, data curation. **Robert P. Takes:** data curation, writing – review and editing. **Gyorgy B. Halmos:** data curation, writing – review and editing. **Dominique V. C. de Jel:** data curation, writing – review and editing. **Jan‐Jaap Hendrickx:** data curation, writing – review and editing. **Richard Dirven:** writing – review and editing, data curation. **Martin Lacko:** data curation, writing – review and editing. **Lauretta A. A. Vaassen:** data curation, writing – review and editing. **Alexander J. M. van Bemmel:** writing – review and editing, data curation. **Reinoud Klijn:** data curation, writing – review and editing. **Marjolijn A. E. Oomens:** data curation, writing – review and editing. **Hossein Ghaeminia:** data curation, writing – review and editing. **Leonora Q. Schwandt:** data curation, writing – review and editing. **Christiaan A. Krabbe:** data curation, writing – review and editing. **Annemarie Vesseur:** data curation, writing – review and editing. **Rolf Bun:** data curation, writing – review and editing. **Thomas J. W. Klein Nulent:** data curation, writing – review and editing. **Jeroen C. Jansen:** data curation, writing – review and editing.

## Consent

Dutch law allows the use of extractions of electronic health records for research purposes under certain conditions. According to this legislation, obtaining neither informed consent from patients nor approval by a medical ethics committee is obligatory for this kind of observational studies containing no directly identifiable data (Dutch Civil Law, Article 7:458).

## Conflicts of Interest

The authors declare no conflicts of interest.

## Supporting information


**Table S1:** odi70082‐sup‐0001‐TableS1.docx.

## Data Availability

The data that support the findings of this study are available on request from the corresponding author. The data are not publicly available due to privacy or ethical restrictions.

## Appendix

Dutch Head and Neck Audit ‐ Oral Cavity Collaborator Group: Robert (R. J. J.) van Es, MD, PhD, Department of Head and Neck Surgical Oncology, UMC Utrecht Cancer Center, Utrecht; Guido van den Broek, MD, PhD, Department of Otorhinolaryngology, Head and Neck Surgery, Radboud University Medical Center, Nijmegen; Robert (R. P.) Takes, MD, PhD (ORCID 0000‐0003‐4784‐0499), Department of Otorhinolaryngology, Head and Neck Surgery, Radboud University Medical Center, Nijmegen; Gyorgy (G. B.) Halmos, MD (ORCID 0000‐0003‐2460‐2260), Department of Otorhinolaryngology/Head and Neck Surgery, University Medical Center Groningen, University of Groningen, Groningen; Dominique (D. V. C.) de Jel, MD (ORCID 0000‐0001‐8159‐0089), Department of Otorhinolaryngology/Head and Neck Surgery, University Medical Center Groningen, University of Groningen, Groningen; Jan‐Jaap (J.J.) Hendrickx, MD, PhD, Department of Otorhinolaryngology, Head and Neck Surgery, Academic Medical Center, University of Amsterdam; Richard (R.) Dirven, MD, PhD, Department of Head and Neck Oncology and Surgery, Netherlands Cancer Institute/Antoni van Leeuwenhoek, Amsterdam; Martin (M.) Lacko, MD, PhD (ORCID 0000‐0003‐2868‐4822), Department of Otorhinolaryngology and Head and Neck Surgery, GROW School for Oncology and Reproduction, Maastricht University Medical Center, Maastricht; Lauretta (L. A. A.) Vaassen, MD, PhD (ORCID 000‐0002‐1408‐6146), Department of Cranio‐maxillofacial surgery, GROW School for Oncology and Reproduction, Maastricht University Medical Center, Maastricht; Alexander (A. J. M.) van Bemmel, MD (ORCID 0000‐0003‐0953‐8408), Department of Otorhinolaryngology and Head and Neck Surgery, Medisch Spectrum Twente, Enschede; Reinoud (R.) Klijn, MD, PhD, Department of Oral and Maxillofacial and Head and Neck Surgery, Medisch Spectrum Twente, Enschede; Marjolijn (M. A. E.) Oomens, MD (ORCID 0000‐0003‐1526‐559X), Department of Oral and Maxillofacial Surgery, Rijnstate Hospital, Arnhem; Hossein Ghaeminia, MD, PhD, Department of Oral and Maxillofacial Surgery, Rijnstate Hospital, Arnhem; Noortje (L. Q.) Schwandt, MD, PhD, Department of Oral and Maxillofacial Surgery, Medical Center Leeuwarden, Leeuwarden; Christiaan (C. A.) Krabbe, MD, PhD, Department of Oral and Maxillofacial Surgery, Medical Center Leeuwarden, Leeuwarden; Annemarie Vesseur, MD (ORCID 0000‐0002‐6702‐4360), Department of Otolaryngology, Head and Neck Surgery, Elisabeth Tweesteden Ziekenhuis Tilburg, Tilburg; Rolf Bun, MD, PhD, Department of Oral and Maxillofacial Surgery/Head and Neck Oncology Noordwest Hospital group, Alkmaar en Dijklander Hospital, Hoorn; Thomas (T. J. W.) Klein Nulent, MD (ORCID 0000‐0003‐3597‐4334), Department of Oral and Maxillofacial Surgery, Haaglanden Medical Center, The Hague; Jeroen (J. C.) Jansen, MD, PhD (ORCID 0000‐0002‐3955‐0152), Department of Otorhinolaryngology, Head and Neck Surgery, Leiden University Medical Center, Leiden.
