# Supplementary material for: Preoperative Staging in Oral Cavity Cancer: Nationwide Practice and Concordance With Pathology
Source: Oral Dis. 2025 Aug 31;32(2):395–404. doi: 10.1111/odi.70082 (PMC13077016; doi:10.1111/odi.70082)
Supplement: Supplementary file 1 — Table S1: odi70082‐sup‐0001‐TableS1.docx. [file ODI-32-395-s001.docx]

**Table S1 – Regression analysis**

|  |  | Accuracy T1 versus T234 | | | | Accuracy T12 versus T34 | | | | Accuracy T4 versus T123 | | | |
| --- | --- | --- | --- | --- | --- | --- | --- | --- | --- | --- | --- | --- | --- |
| Characteristic | N | Event N | OR | 95% CI | p | Event N | OR | 95% CI | p | Event N | OR | 95% CI | p |
| Female sex | 2,458 | 2095 | 1.00 | 0.80, 1.25 | 0.97 | 2180 | 0.97 | 0.76, 1.25 | 0.81 | 2289 | 0.85 | 0.62, 1.16 | 0.31 |
| Age >= 70 years | 2,458 | 2095 | 1.04 | 0.83, 1.30 | 0.75 | 2180 | 1.05 | 0.82, 1.36 | 0.68 | 2289 | 1.00 | 0.73, 1.37 | 0.98 |
| BMI >25 kg/m2 | 2,253 | 1905 | 0.86 | 0.68, 1.08 | 0.19 | 1981 | 1.01 | 0.78, 1.30 | 0.96 | 2088 | 1.32 | 0.96, 1.82 | 0.087 |
| Histology Non-PCC | 2,301 | 1964 | 1.44 | 0.90, 2.47 | 0.14 | 2033 | 0.90 | 0.57, 1.49 | 0.68 | 2137 | 0.76 | 0.45, 1.37 | 0.34 |
| Non-academic hospital | 2,458 | 2095 | 1.13 | 0.89, 1.46 | 0.32 | 2180 | 1.14 | 0.86, 1.51 | 0.37 | 2289 | 1.33 | 0.93, 1.95 | 0.11 |
|  |  | Accuracy N0 versus N123 | | | |  |  |  |  |  |  |  |  |
| Characteristic | N | Event N | OR | 95% CI | p |  |  |  |  |  |  |  |  |
| Female sex | 1,746 | 1350 | 0.96 | 0.77, 1.20 | 0.73 |  |  |  |  |  |  |  |  |
| Age >= 70 years | 1,746 | 1350 | 1.23 | 0.97, 1.55 | 0.082 |  |  |  |  |  |  |  |  |
| BMI >25 kg/m2 | 1,711 | 1326 | 1.07 | 0.85, 1.35 | 0.55 |  |  |  |  |  |  |  |  |
| Histology Non-PCC | 1,631 | 1254 | 2.20 | 0.94, 6.44 | 0.072 |  |  |  |  |  |  |  |  |
| Non-academic hospital | 1,746 | 1350 | 0.86 | 0.67, 1.11 | 0.24 |  |  |  |  |  |  |  |  |
